# Epidemiology of Lassa Fever and Factors Associated with Deaths, Bauchi State, Nigeria, 2015–2018

**DOI:** 10.3201/eid2604.190678

**Published:** 2020-04

**Authors:** Mohammed A. Abdulkarim, Sufiyan M. Babale, Chukwuma D. Umeokonkwo, Eniola A. Bamgboye, Adebobola T. Bashorun, Auwal A. Usman, Muhammad S. Balogun

**Affiliations:** Gombe State Ministry of Health, Gombe, Nigeria (M.A. Abdulkarim);; Nigeria Field Epidemiology and Laboratory Training Program, Abuja, Nigeria (M.A. Abdulkarim, A.T. Bashorun, A.A. Usman, M.S. Balogun);; Ahmadu Bello University, Zaria, Nigeria (S.M. Babale);; Federal Teaching Hospital, Abakaliki, Nigeria (C.D. Umeokonkwo);; University of Ibadan, Ibadan, Nigeria (E.A. Bamgboye)

**Keywords:** Lassa fever, Lassa virus, viruses, epidemiology, confirmed cases, deaths, logistic models, disease outbreaks, Bauchi State, Nigeria

## Abstract

We report the epidemiology of Lassa fever in Bauchi State, a disease-endemic region, in Nigeria. Since 2015, major increases in Lassa fever attack rate and in the case-fatality rate have occurred in this state. A delay in seeking care by a case-patient for >7 days after symptom onset was the major predictor of death.

In recent years, Lassa fever (LF) outbreaks in Nigeria have become more frequent and larger in magnitude; the outbreak in 2018 was described as the largest in history (*1*). Bauchi State, which had never reported an LF case before 2012, has quickly become one of the high-risk states for LF in this country (*2*). We report LF epidemiology in Bauchi State and identify factors associated with death.

We retrospectively reviewed data for LF cases during January 2015–December 2018 obtained from the platform for integrated disease surveillance and response for Bauchi State. The source of the data has 100% completeness for variables of interest (sociodemographic characteristics, laboratory results, outcome of illness, health facility of admission, date of onset of illness, date care was sought, date of death, first health center, clinical features at initial examination, outcomes of laboratory investigations, and treatment outcomes).

We analyzed data by using Epi Info version 7.2 software (https://www.cdc.gov/epiinfo/support/downloads.html). We calculated frequencies and proportions and examined the relationship between the outcome variable (death) and the risk factors (including sociodemographics) by using the χ^2^ test. We included significant variables (p< 0.1) by bivariate analysis and the biologically plausible ones (sex and age) in an unconditional logistic regression model; α = 0.05 was considered the level of significance.

A total of 368 suspected LF cases were reported in Bauchi State during January 2015–December 2018, of which 76 were confirmed. The mean ± SD age for confirmed case-patients was 30.7 ± 15.8 years, and most (81.6%) case-patients were 15–64 years of age. This age group had the highest age-specific attack rate (1.8 cases/100,000 persons), and patients <5 years of age had the lowest attack rate (0.2 cases/100,000 persons). Most (54.0%) patients were male; the attack rate was 1.2 cases/100,000 persons for male sex and 1.1 cases/100,000 persons for female sex. The overall case-fatality rate (CFR) was 54.0% (41/76) and was highest (66.6%) for persons <5 years of age (Appendix Figure 1). All LF cases were reported from districts contiguously located in the southern parts of the state (Appendix Figure 2).

Overall, more cases were reported in the early and late months of the year compared with the middle months (Appendix Figure 3). The CFR trend for LF showed a major increase from 33.3% in 2015 to 53.3% in 2018 (linear trend χ^2^ 4.8; p = 0.03), and the attack rate increased from 0.1 cases/100,000 persons during 2015 to 0.4 cases/100,000 persons during 2018 (linear trend χ^2^ 14.0; p<0.01). We found by multivariate analysis that a delay in seeking care for >7 days after onset of illness (adjusted odds ratio 6.2, 95% CI 1.40–27.60) or for >24 hours after onset of bleeding (adjusted odds ratio 6.4, 95% CI 1.40–29.44) were independent predictors for dying from LF (Table).

**Table:** Independent predictors of dying from Lassa fever, Bauchi State, Nigeria, 2015–2018*

Variable	No. (%) died, n = 41	No. (%) survived, n = 35	Unadjusted OR (95% CI)	Adjusted OR (95% CI)
Sex				
M	21 (51.2)	20 (48.8)	0.8 (0.32–1.95)	0.8 (0.18–3.70)
F	20 (57.1)	15 (42.9)		
Age group				
Productive	33 (53.2)	29 (46.8)	0.9 (0.26–2.75)	1.2 (0.22–6.68)
Dependent	8 (57.1)	6 (42.9)		
Place of residence				
Rural	27 (48.2)	29 (51.8)	0.4 (0.13–1.19)	0.4 (0.08–2.27)
Urban	14 (70.0)	6 (30.0)		
First place of admission				
Tertiary hospital	27 (46.6)	31 (53.4)	**0.2 (0.07–0.85)**	0.2 (0.02–1.45)
Other	14 (77.8)	4 (22.2)		
Bleeding episode†				
Yes	34 (60.7)	22 (39.3)	2.2 (0.73–6.66)	NI
No	7 (41.2)	10 (58.8)		
Duration between seeking care and onset of illness, d				
>7	25 (73.5)	9 (26.5)	**4.5 (1.69–12.08)**	**6.2 (1.40–27.60)**
<7	16 (38.1)	26 (61.9)		
Duration between seeking care and any bleeding episode, h‡				
>24	25 (80.6)	6 (19.4)	**7.4 (2.21–24.81)**	**6.4 (1.40–29.44)**
<24	9 (36.0)	16 (64.0)		

*Bold indicates significance (p<0.05). NI, not included in a regression model; OR odds ratio. †n = 73 for this variable because of missing values. Variable not included in the regression model (p>0.1). ‡n = 56 for this variable because not all case-patients had a bleeding episode.

This study demonstrated that LF has become a highly fatal disease in Nigeria. With the productive age group being the most affected by LF, its socioeconomic impact in the affected communities should be of concern (*3*). A similar age distribution was reported in a study from the neighboring Plateau State in Nigeria, which reviewed confirmed LF cases reported during 2012–2016 (*4*). However, our findings were different from those for a study from Sierra Leone in 2014, in which children and adolescents were more affected (*5*). This finding was probably caused by a difference in cultural environment between the 2 settings.

The southward geographic distribution of LF cases in the study area might be related to the distinctive Sudan savanna vegetation in that part of Bauchi State, which is characterized by higher annual rainfall (which has been shown to influence the incidence of the disease) compared with the Sahel savanna vegetation in the central and northern parts of Africa (*6**,**7*). Furthermore, the intensive agricultural activities in the southern districts and the common postharvest practice of drying crops in open spaces in these hilly areas probably favor food contamination by the disease vector.

Our finding that a delay in seeking care of >24 hours after onset of bleeding was a strong predictor of death among cases is a concern. LF has some common early symptoms similar to those of other febrile diseases, especially malaria, that are frequently encountered in most LF-endemic settings (*8*). An LF diagnosis is often delayed because health workers suspect these other febrile diseases (*8*). Furthermore, Bauchi State has the highest CFR in Nigeria, nearly double the national average (28.9%) (*2*,*9*). A similarly high CFR was reported in a previous study in Sierra Leone in a region affected by conflicts where the health infrastructure was poor (*5*). Finally, the designated LF treatment center in Bauchi State lacks adequately trained personnel and other essential resources to effectively manage complications once they occur. If one considers that a delay in seeking care has been demonstrated to be a predictor of death in this study, the high CFR in Bauchi State could have been lower if the treatment center was better equipped or if cases could be diagnosed earlier.

Appendix Additional information on the epidemiology of Lassa fever and factors associated with deaths, Bauchi State, Nigeria, 2015–2018.
