# Supplementary material for: Epidemiology of Lassa Fever and Factors Associated with Deaths, Bauchi State, Nigeria, 2015–2018
Source: Emerg Infect Dis. 2020 Apr;26(4):799–801. doi: 10.3201/eid2604.190678 (PMC7101116; doi:10.3201/eid2604.190678)
Supplement: Appendix — Additional information on the epidemiology of Lassa fever and factors associated with deaths, Bauchi State, Nigeria, 2015–2018. [file 19-0678-Techapp-s1.pdf]

Article DOI: https://doi.org/10.3201/eid2604.190678

Epidemiology of Lassa Fever and Factors Associated with Deaths, Bauchi State, Nigeria, 2015–2018

Appendix

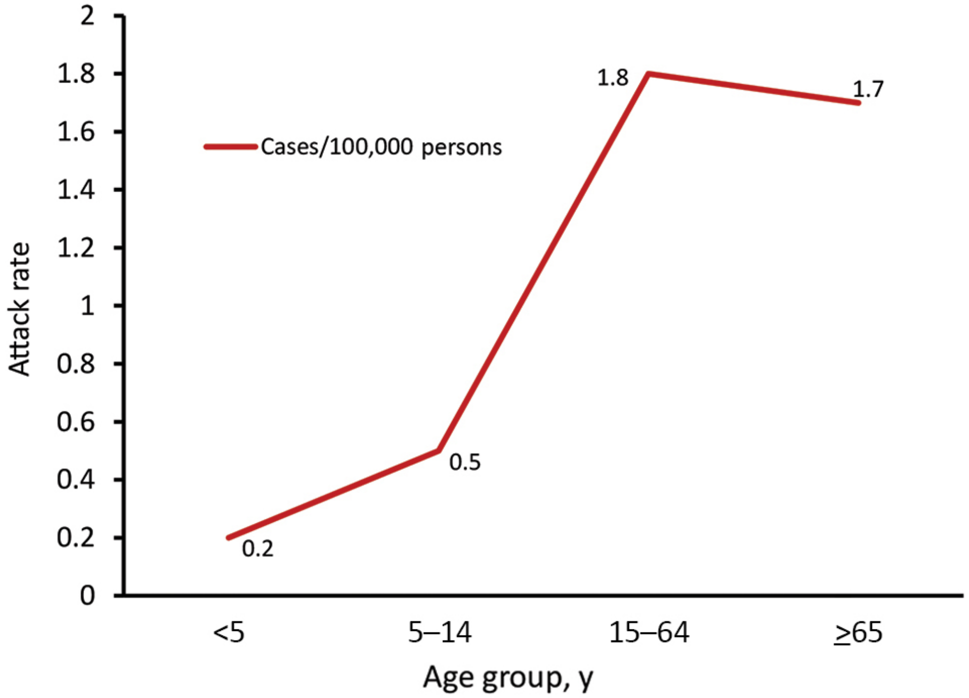

Appendix Figure 1. Age-specific attack rate of Lassa fever in Bauchi State, Nigeria, 2015–2018.

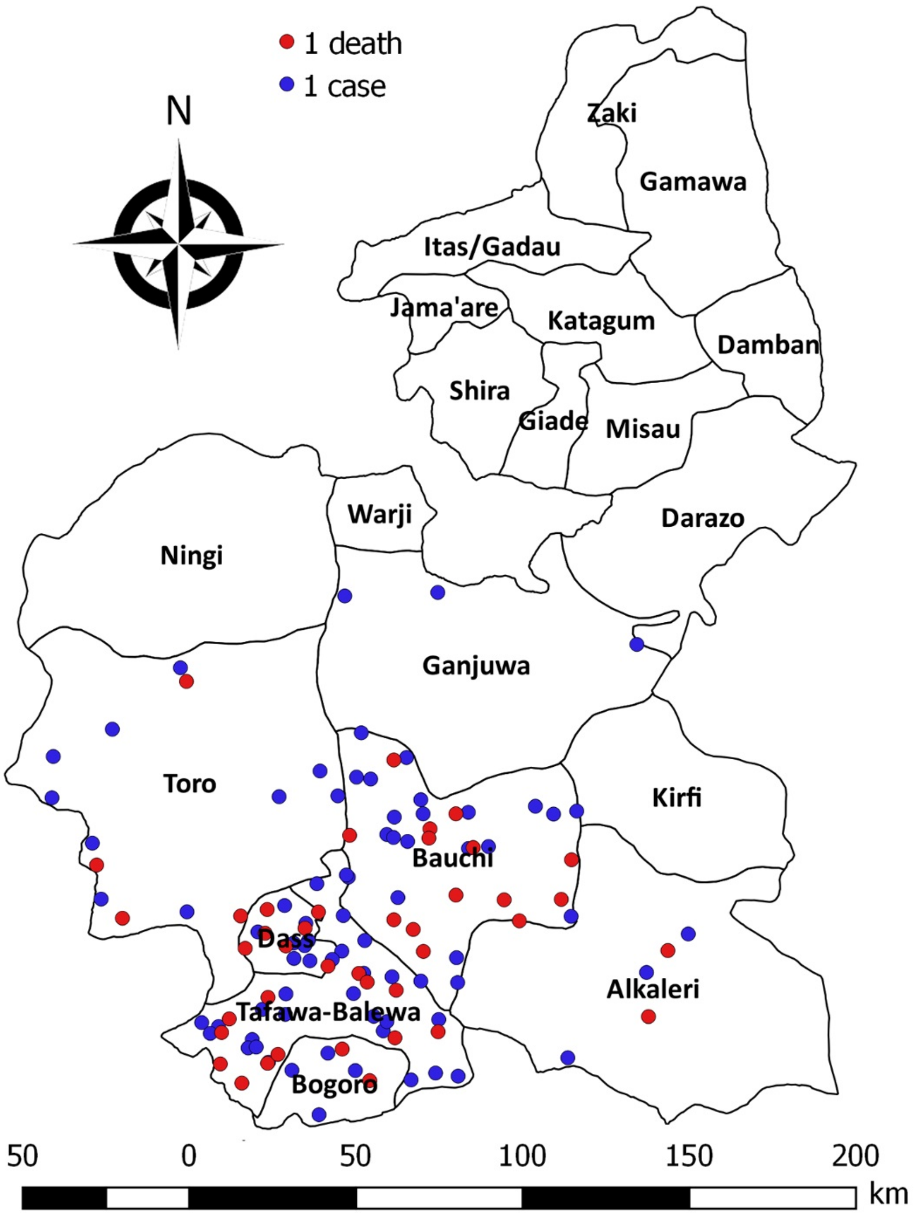

Appendix Figure 2. Distribution of Lassa fever cases in local government areas of Bauchi State, Nigeria, 2015–2018.

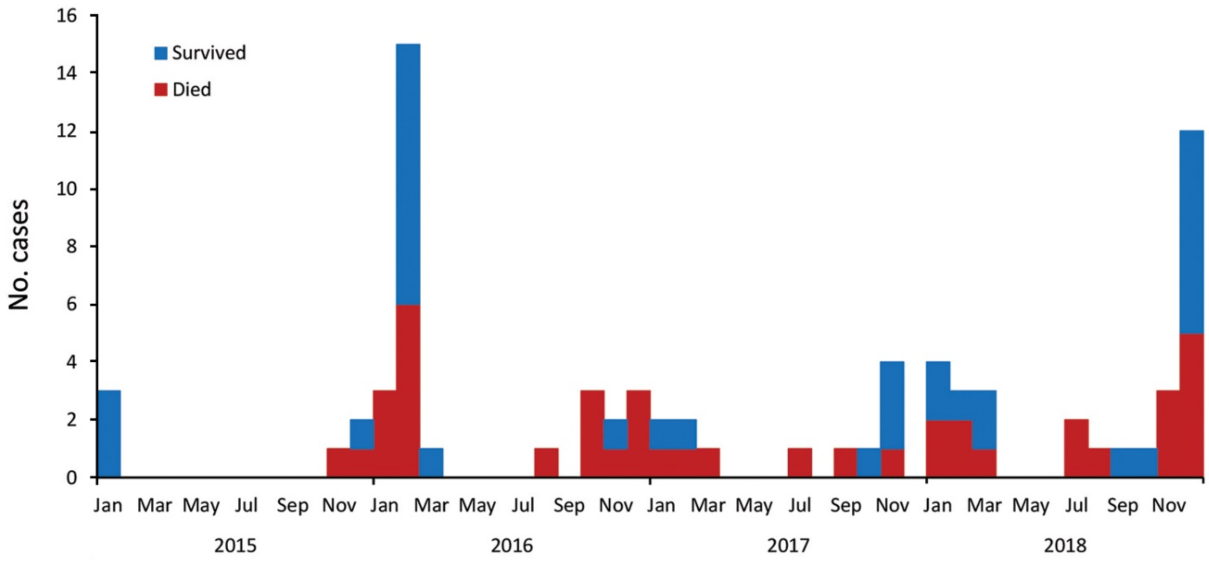

Appendix Figure 3. Lassa fever cases and deaths, Bauchi State, Nigeria, 2015–2018.
